# Beyond traditional training: Integrating data from semi-immersive VR dual-task intervention in Parkinsonian Syndromes. A study protocol

**DOI:** 10.1371/journal.pone.0294199

**Published:** 2024-02-01

**Authors:** Francesca Bruni, Valentina Mancuso, Chiara Stramba-Badiale, Marco Stramba-Badiale, Giuseppe Riva, Karine Goulene, Pietro Cipresso, Elisa Pedroli

**Affiliations:** 1 Faculty of Psychology, eCampus University, Novedrate, Italy; 2 Applied Technology for Neuropsychology Lab, IRCCS Istituto Auxologico Italiano, Milan, Italy; 3 Department of Geriatrics and Cardiovascular Medicine, IRCCS Istituto Auxologico Italiano, Milan, Italy; 4 Human Technology Lab, Catholic University of the Sacred Heart, Milan, Italy; 5 Department of Psychology, University of Turin, Turin, Italy; 6 Istituto Auxologico Italiano, IRCCS, Unit of Neurology and Neurorehabilitation, San Giuseppe Hospital Piancavallo, Verbania, Italy; Transilvania University of Brasov: Universitatea Transilvania din Brasov, ROMANIA

## Abstract

Completing cognitive and motor tasks simultaneously requires a high level of cognitive control in terms of executive processes and attentional abilities. Most of the daily activities require a dual-task performance. While walking, for example, it may be necessary to adapt gait to obstacles of the environment or simply participate in a conversation; all these activities involve more than one ability at the same time. This parallel performance may be critical in the cognitive or motor load, especially for patients with neurological diseases such as Parkinsonian Syndromes. Patients are often characterized by a crucial impairment in performing both tasks concurrently, showing a decrease in attention skills and executive functions, thus leading to increased negative outcomes. In this scenario, the accurate assessment of the components involved in dual-task performance is crucial, and providing an early specific training program appears to be essential. The objective of this protocol is to assess cognitive and motor components involved in dual-task performance and create a training program based on ecological activities focusing on executive and motor functions. Thus, we will employ Virtual Reality to provide semi-immersive, multisensory, ecological, standardized, and realistic experiences for rehabilitative purposes in patients with Parkinsonian Syndromes, considering its high prevalence in aging and the incidence of motor and cognitive dysfunctions in this population. Moreover, we propose to integrate the great amount of different data provided by dual-task and Virtual Reality system, using machine learning techniques. These integrations may increase the treatment’s reliability in terms of better prognostic indexes and individualized training.

## Introduction

One of the most important challenges that social and health systems are facing today is linked to the phenomenon of population aging. The current conditions underline the importance of responding to the constant increase in elderly demand, suggesting new solutions to the problems deriving from the physiological condition of older people, in an effort to better meet their requirements [1,2]. Aging could be often related to impairments in motor and cognitive functioning, which can affect various aspects of daily life depending on the degree of decline, with a consequent possible impact on the quality of life. Although the cognitive and motor aspects have always been treated as separate entities, recent literature has highlighted a relationship between these two aspects, underlining the importance of coordinated interventions to cope with the risks deriving from both impairments [3]. An example of this strong association is locomotion. Locomotion is seemingly a mechanical task, but it couldn’t be reduced to an easy action composed of a series of repeated movements. This action involves the need to simultaneously manage gait performance and one or more cognitive tasks, depending on environmental variability [4,5]. In daily-life activities, it may be necessary to adapt our gait to the obstacles of the environment or simply participate in a conversation; all these activities require us to perform simultaneous tasks. These examples highlight how a complex set of tasks are involved simultaneously in everyday motor abilities. Prupetkaew and colleagues (2019) [6] also conducted research examining the effect of cognitive and motor demand in a free-living environment and they observed alteration of motor patterns while using a phone simultaneously.

A crucial factor in motor performance are executive functions, as highlighted by recent literature [7]. Patients with Parkinsonian Syndromes provide an example of this relationship since they have a significant impairment in their movement capacity effectively and spontaneously [8], a process that is supported by a cognitive load on executive functions. Moreover, evidence stresses the relationship between aging and an altered pattern in terms of motor abilities and cognition that may be affected by different routine abilities with a possible consequence on the quality of life [8]. It is worth mentioning that motor impairments are one of the prevalent features among older adults with neurological disease and one of the most predictors of negative motor outcomes such as falls [3–9]. An increase in the risk of falls derives from the difficulty of carrying out both, motor and cognitive tasks simultaneously, a skill that could be compromised in people who often show a decrease in attention and executive functions [5–10]. In fact, since its initial description, the clinical diagnosis of Parkinsonian Syndromes has primarily focused on motor syndrome. However, nonmotor manifestations are present in most patients and can often predominate the clinical presentation. Moreover, although the rate of progression varies, it is typically quicker in people with late onset and who develop the postural instability gait difficulty form of the disease [11]. *Based on these clinical differences we decided to not consider the early onset of disease*.

A sensitive method for detecting the effect that cognitive load has on gait is the so-called dual-task (DT) paradigm [3], i.e. walking/cycling while performing a secondary task. In recent years, executive function, a crucial cognitive resource for peak performance, has recently evolved into the standard method for evaluating the relationship between cognition and motor skills [12,13]. Executive functions are integrative functions that include both cognitive and behavioural components necessary for effective, targeted actions and to control attentional resources, which are necessary to manage the autonomous activities of daily life [5]. According to recent studies, the DT strategy also seems to be a successful method for concurrent intervention on both cognitive and motor performance [14,15]. In particular, the important contribution of high-order cognitive systems in motor control would make the DT an effective training for reducing risks deriving from ineffective motor patterns. Many studies highlighted the positive potential of combined interventions in decreasing motor difficulties, global function, activity of daily life, and mood in healthy aging and patients [16,17], but further research is needed.

Virtual reality (VR) appears as a powerful tool for the assessment and rehabilitation of aging, and several tools have been successfully created [18–21]. VR is an innovative technology that artificially simulates lifelike experiences to provide users the genuine impression of being inside the real-world [22]. Objects are also depicted in a dynamic, consistent, and accurate realistic way enabling both monitoring of laboratory measurements and naturalistic observation of actual conditions. The possibility to reproduce ecological, realistic, and life-like environments and to create engaging and motivating situations for patients, leads to higher acceptance and adherence rates providing better rehabilitation results. VR can be distinguished as non-immersive, semi-immersive, and immersive according to the device employed during the interaction. Simpler technology, such a desktop PC, employs non-immersive methods to show environments on a single screen; semi-immersive technologies, like the cave automatic virtual environment (CAVE) or large screens, surround the viewer with colossal projections on the walls or screens; fully immersive devices, like Head Mounted Displays (HMDs), for example, are completely immersive systems that separate the user from external world stimuli and offer a full simulated experience, complete with a stereoscopic picture that responds to the user’s head motions [23]. We decided to structure this intervention through CAVE. This technology, among the different types of virtual reality, is a tool that makes it possible to achieve a good level of immersiveness and at the same time allows the motor domains to be stimulated as well. This possibility could be not as easy with wearable virtual reality tools such as the visor. As suggested by Riva and colleagues [24], individuals may benefit from the VR experience and increase their engagement which may determine better outcomes, throughout challenging and interactive tasks. The use of VR for cognitive and motor rehabilitation has already been widely demonstrated by neuroscientists suggesting that it is an appropriate approach in the field of rehabilitation in aging. It has been employed in the past years in motor training with positive results on gait and cognition [25–27], and many different types of interventions are used among neurological patients [21], however, most of them were focused exclusively on gait performance (e.g. strength training and balance exercise). This gap in the research opens new opportunities to develop innovative and standardized training tapping both motor and cognitive aspects simultaneously by means of new technological devices. Furthermore, by providing the patient with immersive tasks, they could have multisensory, ecological, standardized, and realistic experiences during the training.

Another important goal in the recent panorama is the issue of personalization. The possibility to customize interventions based on the specific patients’ need is the aim of the growing scenery in medicine. An increasing number of studies recommend a personalized approach instead of a standard procedure that considers patients in their homogeneity whereas each one is unique despite its pathology [28,29]. To respond to the demand of cutting-edge requirements, we propose a novel approach to forecast which motor or cognitive indicators are most revelatory of long-term maintenance of any improvements obtained. The aim is to verify whether the proposed treatment can be effective in the population according to their clinical characteristics. Estimating the treatment effect, the so-called Treatment Effect Prediction (TEP), is crucial in disease management because it ensures that patients receive the expected clinical outcomes [30]. Machine Learning (ML) techniques offer a great opportunity; they may allow the selection of the most predictive variables, which will be empirically verified via follow-up, also improved based on the historical data of patients, results of neuropsychological traditional tests, and the information provided by the VR system. Using DT tasks, clinicians dispose of a great amount of data compared to a single one, concerning different cognitive indexes and motor variables; additionally, using VR System, allows combining complex tasks providing an even greater amount of data. Thus, a linear approach could be not sufficient, and it may be necessary a multivariate approach, such as ML. ML may be more informative thanks to data fusion, integrating data in a nonlinear model which proves information about treatment prediction. Data from the cognitive, motor, and DT assessment initial evaluation can be compared to ML clinical data and the great amount of features detected during the combined VR intervention to see whether and how patients’ baseline state influenced and predicted rehabilitation outcomes.

The main aim of the present protocol is to create and validate a VR-based program for patients with motor and cognitive impairment. To assess cognition and physical aspects we propose a specific neuropsychological battery, described below. The secondary aim is to improve the quality of life in a sample of older adults with Parkinsonian Syndromes [31], preventing negative outcomes.

## Experimental design

**Fig 1:**
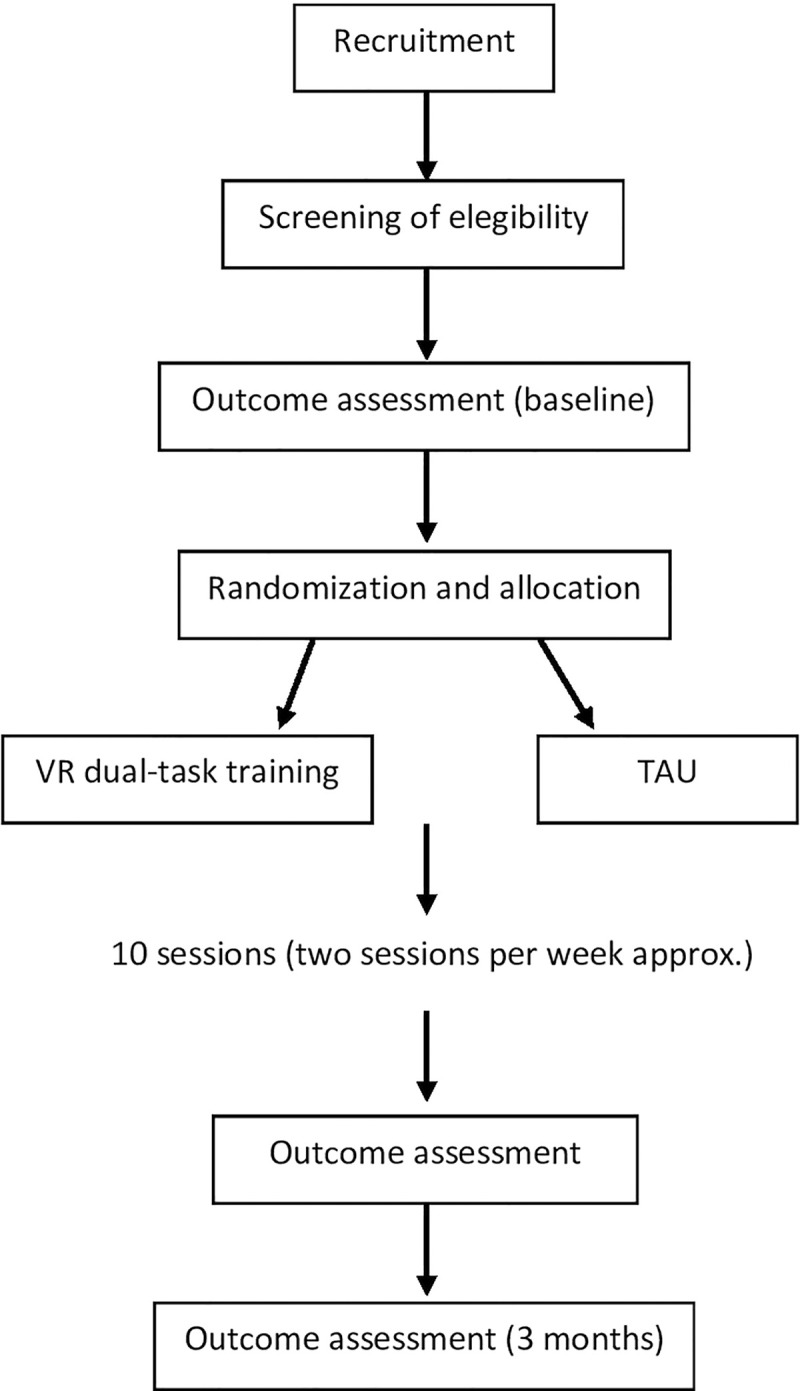
Chart of trial design.

The protocol aims to improve motor and cognitive deficits in patients with Parkinsonian Syndromes. A clinical examination will verify the inclusion and exclusion criteria. The eligible subjects will be assessed, and outcome measures will be collected (t0) by a trained physiotherapist and psychologist. The specialist who assesses participants will not train them. Patients will be then randomly assigned to a control or experimental group using a randomization sequence obtained from the website randomizer.org. The first group will complete the treatment as usual (TAU). Usually, training consists of a series of neuropsychological exercises delivered in paper and pencil modality, and classic motor activity, comprehending aerobic and anaerobic activities delivered thanks to bodyweight exercises on a stationary bike in an ordinary gym. The other one will complete a VR dual-task training protocol. Based on the evidence that exercise programs with shorter session duration and higher frequency may generate the best results [23], we propose 4–5 weeks of training of 10 sessions of 1 hour approximately, two times a week. Completed the 10 biweekly rehabilitation sessions a new assessment will be done (t1). The neuropsychological and motor assessment will be carried out before (t0), immediately after (t1) and 3 months after the end of the 10 sessions (t2) to evaluate the short/medium-term efficacy of the treatment. The chart of the trial design is presented in Fig 1. Patients must attend at least 8 out of 10 rehabilitation sessions as well as all the assessments for the treatment to be considered effective. All participants will sign the written informed consent.

## Materials and equipment

The platform used during the training will be an immersive VR apparatus available at the Department of Geriatrics and Cardiovascular Medicine of the Istituto Auxologico Italiano: Cave Automatic Virtual Environment (CAVE). This system is composed of a room-sized cube in which four stereoscopic projectors (Full HD 3D UXGA DLP) are used to cast a three-dimensional image of the Virtual Environment (VE) onto three walls and the floor. In particular, there are three retro-projected screens (frontal, right, and left) and a direct-projection screen (floor screen). Four infrared cameras are employed to monitor any movements made by 3-D glasses. Active goggles combine the projected images for the right and left eyes to enable the feeling of depth. A tracking optical system is included in CAVE in addition to the visualization tools (VICON). The virtual scene in the CAVE is encoded with depth information, which is recovered and delivered to the eyes via 3-D glasses. The user has the impression that he/she is still moving around the object in 360 degrees because of the different images and viewpoints. As the user moves their head, the image almost spins in real time. An asymmetrical set of markers on both CAVE goggles and an Xbox joystick enable the retrieval of their position and heading in the environment. These data bits are utilized to enable the Xbox joystick to be used as a pointer for interfacing with 2D interactable components (such as buttons) in the CAVE and to modify the user’s point of view, respectively. A cluster system made up of two HPZ620 Graphics Workstations and an Nvidia Quadro K6000 GPU with specific Quadro Sync cards manages all the CAVE features. This technology ensures a true-to-life experience.

Different tools were created to perform several activities in the Cave. Training will be developed based on existing tools, enriching and combining them to create a new training protocol. It will involve different dual-task exercises: the Positive Bike, Rocks, and the Supermarket. They will be proposed in a randomized order within subjects.

- *Positive Bike*. It is an innovative immersive tool proposed by Pedroli et al. (2019a) [19] which consists of a stationary bike placed inside the CAVE in which patients cycle and keep their cycling velocity steady (motor task); concurrently they have to recognize target objects between distractors (cognitive task). The therapist decided the exercise parameters (e.g. time between targets presentation, the target to select, bike velocity) in each session.
- *Rocks*. The exercise, developed by Pedroli et al (2019) [19], originates to train balance. Patients are immersed in a virtual environment resembling a straight dirt road and have to avoid rocks (motor task) they encounter on the way, moving their body in the right or left direction. We will add a cognitive part: while moving subjects will have to declare their direction (e.g. they will say ‘right’ if rocks move on the right and ‘left’ if they move on the left).
- *Supermarket*. This exercise takes place in a virtual supermarket, as proposed by Pedroli et al. (2018) [32]. It aims to train executive functions: users have to move in the shop using an Xbox controller and they must buy several products following precise rules. Ten different tasks with increasing difficulty are available. To create a dual-task, while shopping, we will add a motor task consisting of a walk in place with a metronome.

**Fig 2:**
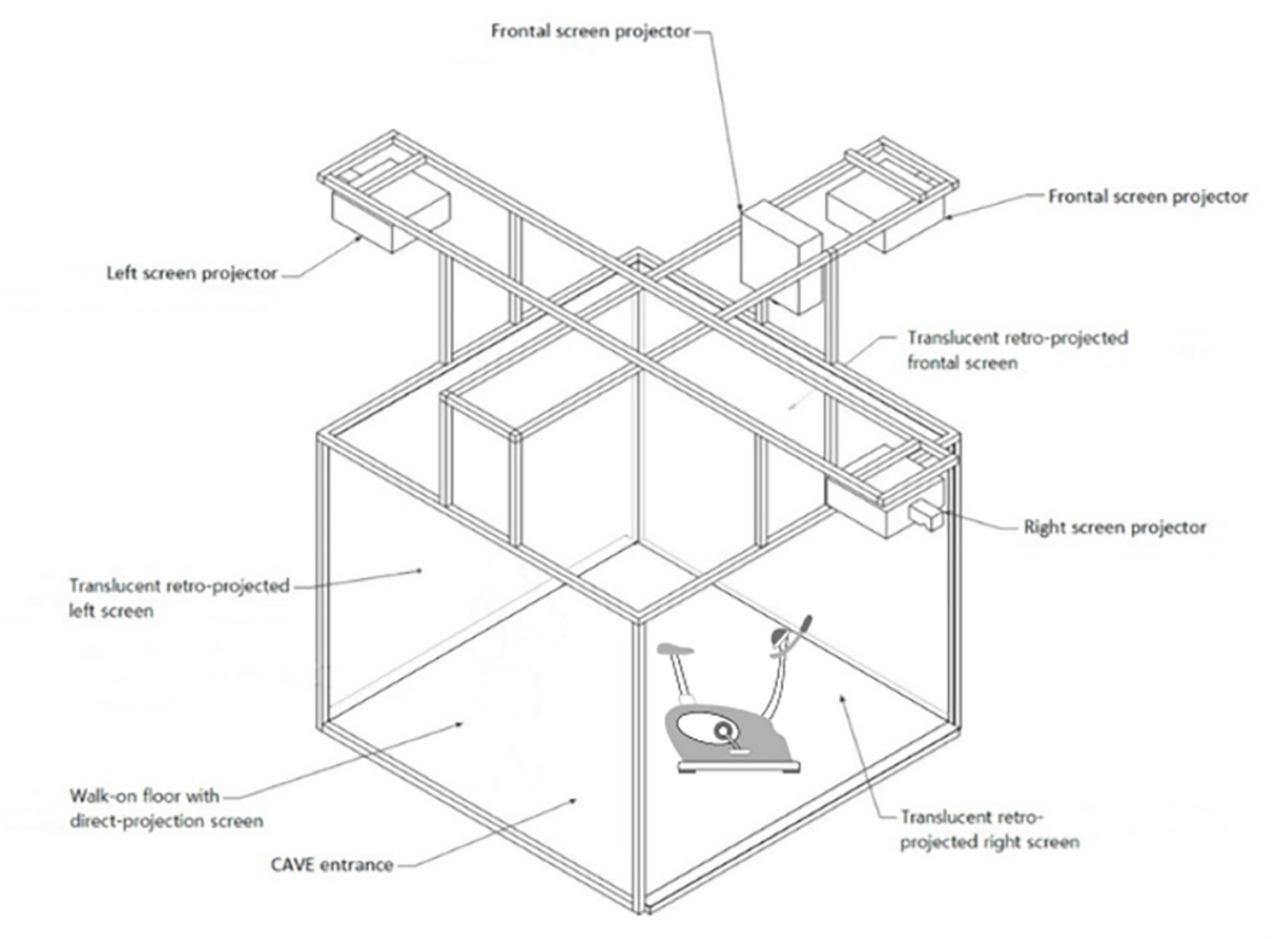
A schematic representation of the VR system, CAVE, with the stationary bike.

All cited VEs were created utilizing Unity 3D and the MiddleVR Unity plug-in. This plug-in enables the communication between the Unity application and all the CAVE system components, allowing for the projection of scenes onto the CAVE walls and the use of motion data from the VICON system as inputs. The parts of the system are highlighted in Fig 2.

## Participants

Participants will be volunteers of both sexes, aged 65 or over (without maximum age limitation), and with Parkinsonian Syndromes as classified by Williams and Litvan (2013) [31]. The eligibility criteria will require the participants to have an MMSE [33,34] score between 30 and 24 and all participants will have normal or corrected-to-normal vision. Exclusion criteria will be invalidating internist, psychiatric, neurological conditions, presence of depression or anxiety without medications and hemianopsia or hemiplegia, severe physical or functional limitations impeding physical activity, and recurrent vertigo. The presence or absence of these criteria will be assessed during the initial clinical assessment performed by a physician. The final sample will be composed of 45 patients; to achieve this goal, at least 50 subjects will be assessed. To evaluate the size of the samples, we used a Sample Size Calculation (Power Analysis) using the software GPower*3. Based on data from Killane et al. [22], with an effect size of 0.43, alpha of 0.05, and 80% power and we estimated the minimum of subjects to be included in the rehabilitation experiment.

## Outcome measure

Participants will be assessed to provide information regarding their motor and cognitive abilities, as well as their capacity to perform the mentioned actions simultaneously. We will include evaluation scales related to the functional aspects such as the Tinnetti Balance Scale, the Equiscale, and the Time Up and Go Test (TUG). The Tinetti Balance Scale [35] is the gold standard scale for balance evaluation. It is a simple clinical test consisting of 14 items with a score out of 28. The therapist evaluates patients’ performance in some activities concerning balance, gait, and risk of falling (i.e. standing up, walking, standing down, etc.). The higher the score, the better the performance. The Equiscale [36] includes three subdomains of standing up, resistance to external perturbations, and resistance to self-induced perturbation in a real-life performance, for example, the therapist evaluates the ability to lean forward, sit up, etc. The Timed Up and Go Test (TUG) [37] examine balance, gait speed, and functional abilities required to performance of basic activities of daily living; it measures the time that the subject takes to get up from a standard chair, walk three meters, turn around and go back to sitting down. The highest the time the worse the performance. The optimal cut-off value would be considered from 10 to 33 seconds.

We will also evaluate cognitive domains; the general cognitive status with the MMSE and the executive functions domain with the Frontal Assessment Battery (FAB), the Tower of London (ToL), the Trail Making Test (TMT), and the Stroop Test. The MMSE [33,34] is a brief test that screens the global functioning including temporal and spatial orientation, memory, attention, language, and apraxia. FAB [38] is a screening test composed of six cognitive and behavioural tasks: similarities, phonological verbal fluency, motor series, conflicting instructions, Go-No Go task, and prehension behaviour. The score ranges from 0 to 18. The ToL [39] is traditionally used to assess strategic reasoning, problem-solving, and mental planning in clinical populations. The task consists of moving tree beads to reproduce the target configuration; time and accuracy are evaluated. The TMT [40] measures attention and ability of set-shifting; it is composed of two parts: the first (TMT A) is a searching task, and the second part (TMT B) requires shifting in searching alternatively numbers and letters. During the performance, time is evaluated: the highest time the worst score. The Stroop Test [41] is designed to test the capacity to inhibit cognitive interference, which happens when processing one sensory characteristic interferes with processing another at the same time. It is composed of three parts, for each one the person is instructed to read respectively all the words, colors, and color ink as quickly as possible. A trained physiotherapist and psychologist will perform the assessment to exclude the low reliability of the data.

The ability to engage in both motor and cognitive tasks will be evaluated with the motor and cognitive Time Up & Go Test (TUG) [42] and the Walking and Remembering Test (WART) [42]. Motor and cognitive TUG consists of the previously described motor performance with an adjunct cognitive part requiring the patient to count backward by threes while walking. Similarly, the WART evaluates single and dual-task performance: working memory task while walking. Patients had to walk at their fastest safe speed along a path in both single and dual-task (simultaneous digit span task) conditions. Average walking time was considered.

At last, quality of life will be evaluated using the Italian validation of the Parkinson’s Disease Questionnaire (PDQ-39) [43]. Outcomes information are included in Table 1.

**Table 1:** Outcome measurements.

Test	Outcomes information
*Motor*	
Tinnetti Balance Scale	Balance
Equiscale	Standing and resistance to external and self-induced perception
TUG	Mobility
*Cognitive*	
MMSE	General cognitive status
FAB	Screening of executive function
ToL	Reasoning and problem-solving
TMT	Attention and set-shifting
Stroop Test	Cognitive flexibility and sensitivity to interference
*Dual-task ability*	
Motor and cognitive TUG	Balance and executive functions
WART	Gait and working memory
**Questionnaire**	**Outcomes information**
PDQ-39	Quality of life

Moreover, we will consider outcome variables provided by the CAVE system during the DT training such as the time, accuracy, velocity, etc. performing each exercise.

## Data analysis

We will realize a mixed design trial (2x3) with population training (VR vs. TAU) as a between variable and assessment of training effect (pre-test, post-test, and follow-up) within variables. A Windows Excel sheet will be used to organize all the data related to demographic and assessment information and an identification code will be assigned to each participant to ensure anonymity of the data. A mixed ANOVA will be performed to investigate interaction effects between and within variables.

Moreover, we will analyze the accuracy during DT training to verify possible correlations with the accuracy measure in the assessment part at t0 and t1. The objective will be to examine if traditional tests reflect trained abilities.

Moreover, we will propose an innovative approach of ML in the rehabilitation field, to estimate the TEP from the collected data during the DT VR experience. Based on our research question and available data we will use a classification algorithm to create a model; we will train and test the model using data to forecast which motor or cognitive parameters are more predictive for the future maintenance of any improvements obtained, based on the model proposed by Shi and colleagues (2019) [44]. We will use one of the best programs for ML analysis, such as Python.

## Discussion

This VR dual-task rehabilitation protocol aims to assess and train motor abilities and executive function in older people characterized by a deterioration in either motor or cognitive execution or even both, capitalizing on the massive amount of information extrapolated from patients during their diagnosis and treatment process, thanks to ML. The final scope is also to improve patients’ quality of life, considering that most daily activities require dual-task abilities. Considering these premises, we evaluate the ability to perform DT by using exercises as similar as possible to what people encounter in daily life. We will develop an innovative tool employing an immersive VR system and we will compare this innovative protocol with a selection of traditional exercises. Based on promising literature on VR and DT training, the presented protocol could be as effective as or more effective than classical rehabilitation. Several studies showed that combining physical and cognitive training is more successful compared to a single physical or cognitive exercise [27]. Moreover, the use of VR seems to be a crucial element to enhance successful and fulfilling rehabilitation [26,45]. VR has an important value in rehabilitation: it provides an intervention program based on the latest technological advances that act on crucial components of daily life. Based on the literature, the presented protocol seems to be promising in training motor and cognition. However, further studies will analyze the usability and the clinical efficacy of the tool, also examining participants’ perceptions of the used technology.

Moreover, recent researchers underline the importance of proposing continuative assistance to contribute to the maintenance of obtained benefits. New challenges and opportunities come from Ambient Assisted Living which can help people at home in many ways, including social integration, health, lowering the risk of cognitive decline, and preventing premature death. This innovation could improve the wellness and health conditions of older adults, allowing the prevention and management of diseases. Nowadays, AAL includes tools such as medication and events reminders [46], monitoring systems for fall detection [47,48], assistive devices in the activities of daily life [49,50], and technologies to promote a healthier lifestyle and socially-active aging [51]. New ideas of application proposed to train motor and cognitive abilities are arising [48,52]. Accordingly, this new protocol might be customized to be used at home, providing a tablet-based tool with a portable cycle ergometer to perform the dual-task exercise. Although this modality could not as immersive as the one provided with the CAVE, it could be an effective solution to promote the continuity of care also in patients that access in hospital for a limited period, providing a presumably safer environment to train them for the dual-task challenges with practical applications [53–55], which reflect ecological situations. In these panoramas, it could be useful to promote joined interventions which include both in-hospital interventions followed by at-home training to retain the improved capabilities.

The use of DT and new technology joined with collecting big data useful to integrate with outcomes provided from the assessment offering more reliable and predictive neuropsychological results compared to the classic paper and pencil tests. The application of ML techniques could optimize individual treatment strategies, helping the transition to personalized, effective, and engaging medicine, built on the individual patient’s needs. In the literature, ML is linked to diagnostic screening methods, with subsequent disease progression analyses [50–52]. Using ML in the rehabilitation field is a new strategy that allows exact predictions on which motor or cognitive measures are more predictive of future maintenance of any improvements obtained. Identifying such aspects could promote the movement away from a one-treatment-suits-all-approach to the development of personalized treatment in the context of the broad spectrum of cognitive training interventions. In this scenario, clinicians could know which patients’ characteristics predict the efficacy of different training modes and what type of training works best. Health professionals could also save patients’ time in the assessment phase by providing training quickly and efficiently. Indeed, interventions that are specific and well-timed are required to maintain cognitive performance and decrease the progression of dementia and physical decline. For example, early detection of gait abnormalities allows for the timely identification of not just motor risk but also cognitive loss, allowing for appropriate intervention [53]. Further, in some cases, it could be more effective to use vertical therapy than a traditional dual-task or a VR dual-task, providing long-term improvement in rehabilitative outcomes. Therefore, ML can augment the ability of healthcare providers to improve patient care, deliver accurate diagnoses, optimize treatment plans, inform decisions, or allocate resources within health systems [56,57].

## Limitation and conclusion

Limitations apply to the current work as well. CAVE systems are not as common in healthcare settings because they are expensive technology solutions and need some space. There are currently commercial solutions available at low cost, such as the Head Mounted Display (HMD), much more portable and with a greater capacity for immersion and presence. However, the CAVE among the different types of VR is a tool that makes it possible to achieve a good level of immersion allowing motor exercises without limitations. This possibility is not as easy with other VR tools, such as HMD, which could limit the users’ movements. Users wear their HMD and they do have not a complete visual of anything that happens outside the visor; this could generate feelings of apprehension and they may reduce their movements. Moreover, the CAVE offers the possibility to recreate a more realistic dual-task situation: users may view their real bicycle while cycling in a park for example, improving their engagement and immersion in the task.

Another limitation concerns the possible negative effects of using VR, i.e., cybersickness. Cybersickness is a potential result of the intensive visual and motion cuing when encountering a virtual environment, and it is defined as a set of symptoms including nausea, weariness, headache, strain, postural instability, and vomiting [58]. However, we take into account some parameters which could minimize these consequences, such as considering realistic movements to match sensory expectations, creating a clear, steady horizon environment with reference points, and limiting the time of exposure [58].

Despite some limitations, the project arises from an emerging question in the research field and aims to contribute by providing several benefits also on services and the community. On one hand, the protocol aims to ameliorate autonomy and quality of life in a sample at risk of frequent falls and cognitive impairment; on the other hand, the project might improve the Italian healthcare system by providing innovative treatment based on recent advances in neuroscientific and technological research, opening new opportunities in this field.
